# Melatonin, Its Metabolites and Their Interference with Reactive Nitrogen Compounds

**DOI:** 10.3390/molecules26134105

**Published:** 2021-07-05

**Authors:** Rüdiger Hardeland

**Affiliations:** Johann Friedrich Blumenbach Institute of Zoology and Anthropology, University of Göttingen, Bürgerstr. 50, D-37073 Göttingen, Germany; rhardel@gwdg.de

**Keywords:** AMK, carbonate radicals, melatonin, nitration, nitric oxide, nitrosylation, peroxynitrite

## Abstract

Melatonin and several of its metabolites are interfering with reactive nitrogen. With the notion of prevailing melatonin formation in tissues that exceeds by far the quantities in blood, metabolites come into focus that are poorly found in the circulation. Apart from their antioxidant actions, both melatonin and *N*^1^-acetyl-5-methoxykynuramine (AMK) downregulate inducible and inhibit neuronal NO synthases, and additionally scavenge NO. However, the NO adduct of melatonin redonates NO, whereas AMK forms with NO a stable product. Many other melatonin metabolites formed in oxidative processes also contain nitrosylatable sites. Moreover, AMK readily scavenges products of the CO_2_-adduct of peroxynitrite such as carbonate radicals and NO_2_. Protein AMKylation seems to be involved in protective actions.

## 1. Introduction

Melatonin is mostly perceived as a highly pleiotropic regulator molecule that controls or modulates numerous physiological processes [1,2]. Originally discovered as a hormone of the pineal gland, it is now known to be produced in, perhaps, all tissues of the body [3,4]. Differences between pineal and extrapineal melatonin, concerning chronobiotic and nonchronobiotic actions, higher amounts produced in extrapineal sites with low rates of release have been extensively summarized elsewhere [1,4]. Mitochondrial synthesis of melatonin [4,5,6] has, again, changed our understanding of this molecule, which exceeds by far the earlier chronobiological conceptions. This view is well in accordance with the previously identified bacterial origin of this molecule [7]. Moreover, melatonin has become known as an antioxidant that upregulates protective enzymes of redox metabolism and scavenges reactive oxygen species (ROS) [8,9,10,11,12,13]. In fact, the protective function of melatonin exceeds these roles. Even though major findings concerning the detoxification of reactive nitrogen (RNS) are known since several years, the significance of this aspect is often poorly considered by researchers. Especially from a pathophysiological point of view, nitric oxide and its biochemical derivatives deserve particular attention and concern numerous pathologies. These compounds are of relevance to all kinds of high- and low-grade inflammation, neuronal overexcitation, as well as traumatic or ischemic injuries of brain and other organs [14,15,16,17,18,19,20,21,22,23]. Moreover, NO levels are of fundamental importance to mitochondrial function, and rises of NO and its derivative, peroxynitrite, are involved in processes of dysfunction, damage and cell death [17,19,20,21,22,24].

A rather intriguing aspect of melatonin’s protective actions is related to the participation of its metabolites. These exceed those which are formed for purposes of melatonin clearance by CYP subforms and conjugating enzymes. Others have been shown to be relevant to the metabolism in tissues, as most clearly documented in the skin [25,26,27]. Already the number of those which are nonenzymatically formed is remarkable and their chemical properties are divergent, in terms of reactivity, stability and spectrum of actions [28,29,30,31].

It is the aim of this article to outline the effects of melatonin and its metabolites on biosynthesis, elimination and conversion of RNS, with a particular focus on properties and actions of the nonindolic metabolites, which have been often left out of sight when discussing the protective potential of melatonin.

However, the multiple protective actions against NO and its derivatives should not be misinterpreted. As a physiologically important regulator, NO is a primarily beneficial and necessary agent, e.g., in terms of vascular control and communication within the central nervous system. In fact, NO is of value in cardiovascular protection [32,33,34] and in mitochondrial function [20]. Problems arise by overshooting NO formation, especially under conditions of inflammation, under which melatonin can correct unfavorable deviations.

## 2. Nitric Oxide, Its Congeners and Reactive Products

### 2.1. Formation of NO Redox Congeners

Nitric oxide is formed by endothelial, neuronal and inducible NO synthases (eNOS, nNOS, iNOS) as a free radical (^•^NO). However, two redox congeners also exist, namely, NO^+^ and NO^−^ (in biological material, according to pK_a_, present in its protonated form, HNO) [35,36]. The nitrosonium cation, NO^+^, is easily formed from various nitrosothiols, such as S-nitrosocysteine and S-nitrosoglutathione, compounds that are physiologically generated at elevated ^•^NO levels. Moreover, it can be produced by iron- and copper-containing metalloproteins. For instance, binding of ^•^NO to Fe^III^ of hemin in methemoglobin or cytochromes yields complexes [Hb(Fe^III^)^•^NO ↔ Hb(Fe^II^)NO^+^] that either redonate ^•^NO [e.g., Hb(Fe^III^)^•^NO → Hb(Fe^III^) + ^•^NO] or releases NO^+^ [e.g., Hb(Fe^II^)NO^+^ → Hb(Fe^II^) + NO^+^]. Moreover, the NO metabolite nitrite is reduced by deoxyhemoglobin, thereby forming the Hb(Fe^III^)^•^NO complex, which releases, as Hb(Fe^II^)NO^+^, the nitrosonium cation [37]. Hence, it may not be surprising that NO^+^ was reported to be physiologically formed in arterioles, capillaries and venules [38], Notably, the contributions of NOS isoenzymes to ^•^NO formation differed between these vessels [38]. Another source of NO^+^ is MnSOD (manganese superoxide dismutase), which also dismutates, in a side reaction, 2 ^•^NO to NO^+^ and NO^−^ (the latter being soon protonated to HNO) [39].

Apart from this dismutation reaction, biological processes leading to HNO formation are insufficiently understood. To date, there is no generally accepted route of NO^−^/HNO biosynthesis of quantitative relevance [40]. Among processes of presumably minor importance, only a few mechanisms have been identified. For instance, HNO can easily nitrosylate thiols, such cysteine or glutathione, but HNO is not redonated at substantial rates by the reverse reaction but can be generated by interaction of a nitrosothiol with another thiol [RS-NO + RSH → RSSR + HNO] [40]. Another possibility consists in the hydrolytic decay of nitrosocarbonyls (formed by oxidation of, e.g., amino acid amides such as asparagine or glutamine) [R-CO-NO + H_2_O → R-COOH + HNO]. Even though the physiological formation of HNO remains to be further clarified, this compound is of pharmacological interest. On the one hand, HNO has been shown to exert positive inotropic effects via ryanodine receptors in the heart that are distinct from actions of ^•^NO [40,41,42]. Another aspect concerns HNO formation from a drug, cyanamide, which is used as alcohol dehydrogenase inhibitor for the treatment of alcohol intoxication. Cyanamide is converted by catalase [H_2_N-CN + H_2_O_2_ → HNO + CN^−^ + H^+^]. Notably, HNO/NO^−^ is not generated from ^•^NO, as evident from the redox potentials. It is also not formed via binding of HNO to hemin, since it serves as a reductant [Fe^III^ + HNO → Fe^II^-NO + H^+^] [40]. With regard to melatonin, HNO is of interest, because its nitrosylation capacity extends to various indoles, including melatonin [36], as will be outlined in Section 4.

### 2.2. Nitric Oxide by Reduction of Nitrate and Nitrite: External Sources and Metabolite Recycling

In addition to ^•^NO formation by NO synthases, NO can be also produced by reduction of nitrite. On the one hand, nitrate and nitrite are known as metabolites of NO, but can also be taken up from nutrients. Dietary nitrate is mostly concluded to be reduced to nitrite by bacteria present in the oral cavity, whereas nitrite is reduced to NO in the intestine, as part of the enterosalivary nitrate cycle [43,44,45] or by several enzymes in various tissues. A mammalian nitrate reductase has been reported to exist in human and rodent tissues [46]. Nitrite reductase activities have been described for several metalloproteins and metal-containing enzymes known for different functions. These comprise hemoglobin [37,47], myoglobin [48], cystathionine β-synthase [49] and indoleamine 2,3-dioxygenase 1 [48]. Even though the latter enzyme is known for its melatonin-oxidizing property, there is no reason to assume a functional cross-connection in this place. Which NO congener is released by the metalloproteins, depends on the dissociation of the nitroso-iron complex, as outlined above for hemoglobin. The intriguing question of whether melatonin has any influence on nitrate/nitrate reduction has been poorly investigated. As far as nitrate and nitrite have been formed from previously synthesized ^•^NO, one might assume that downregulation of especially iNOS may be followed by lower rates of nitrite reduction. Effects of melatonin on NO of dietary origin are poorly conceivable. Physiological effects of melatonin on nitrate and nitrite reductions are to date only known from plants [50].

### 2.3. Properties of the NO Congeners

The physiological and pathophysiological significance of the three redox congeners, NO^+^, ^•^NO and HNO, has to be judged differently. ^•^NO is, according to actual knowledge, the only redox form that exhibits properties as a signaling molecule. While it is beneficial as a blood flow-enhancing compound [51,52,53,54] and, at low concentrations, also in mitochondrial protection [20], its overproduction in the context of excitotoxicity [18,21,55] and inflammation [16,17,18,19,21,22,23] can be highly detrimental. This concerns especially the mitochondrial effects of high ^•^NO levels, starting with interruption or partial blockades of electron flux by binding to heme and nonheme irons, nitrosylation of protein and nonprotein thiols and, in particular, reactions by derivatives of ^•^NO, as discussed in Section 4. Consequences of these disturbances are enhanced electron dissipation at Complexes I and III, opening of the mitochondrial permeability transition pore and, in the extreme, enduring breakdown of the mitochondrial membrane potential (ΔΨ_mt_), which leads to cell death. Compared to ^•^NO, the effects of NO^+^ and HNO are largely devoid of excitatory and inflammatory signaling, whereas these congeners share several other properties, concerning especially binding to iron and thiol nitrosylation. Insofar, unfavorable actions can be expected by both of them, in particular, at the mitochondrial level. In fact, most actions of NO^+^ were reported to be detrimental and, in fact, caused ΔΨ_mt_ breakdown and apoptosis induction [36,56,57,58,59,60,61,62,63,64,65,66,67,68]. The apoptotic effect of NO^+^ was found to be considerably more rapid that that by ^•^NO [56,57]. Other experiments revealed enhanced degradation of IRP2 (iron regulatory protein 2) by NO^+^ and, additionally, a strong posttranscriptional upregulation of ferritin expression [58,59]. It has also been discussed whether protein S-nitrosylation by NO^+^ may be involved in IRP2 degradation and whether secondarily formed NO^+^ might play a specific downstream role in inflammation [59], although it does not represent a proinflammatory signal molecule such as ^•^NO.

The biological and toxicological relevance of HNO is an intriguing field, which, however, requires more profound insights. In accordance with the above-mentioned statement that the capacity for thiol nitrosylation would suggest a potential for mitochondrial toxicity, cell damage had, in fact, been repeatedly observed, despite some other potentially beneficial effects concerning cardiac function [60,61,62,63]. HNO was even reported to be by far more cytotoxic than ^•^NO, almost as toxic as peroxynitrite [64]. Moreover, HNO was shown to interfere with mitochondrial respiration by inhibiting Complexes I and II [65]. Surprisingly, additional data indicated intramitochondrial formation of HNO [65], however, without demonstrating a convincing chemical mechanism. The above-mentioned route via interaction of a nitrosothiol with another thiol may be a possibility. An alternative may be the earlier suggested reduction of ^•^NO to NO^−^ by ferrocytochrome c [66]. In contrast to the toxicological findings on HNO, this compound had also been discussed as an anti-inflammatory agent [67]. It remains to be clarified whether these concepts are truly controversial or reflect different aspects that appear in different concentration ranges.

### 2.4. Other NO-Derived Reactive Nitrogen Species and Generation of Secondary Radicals

Apart from the three NO redox congeners, various other nitroxides exist and can occur in biological systems. Nitrous oxide (N_2_O) is formed by various bacteria and archaea, but not by vertebrate tissues, although it may appear there by diffusion from the gastrointestinal tract where it is formed by the microbiome. No chemical interactions between N_2_O and melatonin or its metabolites are known. However, ^•^NO_2_ is produced in the vertebrate body and participates in nitration reactions, as will be discussed below. Its formation is possible by the canonical interaction of nitric oxide with oxygen [2 ^•^NO + O_2_ → 2 ^•^NO_2_], but other routes to be outlined subsequently seem to be of higher physiological and pathophysiological relevance. Moreover, combination reactions exist for nitroxides that lead to N_2_O_2_ from 2 ^•^NO and N_2_O_4_ from 2 ^•^NO_2_. However, their biological relevance may be negligible, since the equilibria are far on the side of the monomers.

The situation is considerably different in the case of N_2_O_3_. On the one hand, this compound can be seen the result from a combination of ^•^NO and ^•^NO_2_. Conversely, N_2_O_3_ has been regarded as an ^•^NO donor and a major nitrosylating agent. In fact, nitrosylation of indoles including melatonin by N_2_O_3_ has been described [68,69]. Comparative studies on melatonin nitrosylation showed that N_2_O_3_ was about 50 times more effective than peroxynitrite [69]. Nevertheless, questions arose concerning the relevance of this reaction in vivo. The major objection came from the rapid decay of N_2_O_3_, in biological systems, since it is easily hydrolyzed [N_2_O_3_ + H_2_O → 2 HNO_2_] [70]. Nevertheless, the resulting nitrite, which is anyway a major ^•^NO metabolite, remains to be an active player in the RNS world. Interestingly, nitrite was identified as a relevant compound in the generation of N_2_O_3_, in another pathway that is based on the binding of nitrite to methemoglobin [71,72]. Two possibilities have been discussed. The first one starts with ^•^NO binding to methemoglobin [Hb(Fe^III^)^•^NO ↔ Hb(Fe^II^)NO^+^] and is followed by a combination with nitrite [Hb(Fe^II^)NO^+^ + NO_2_^−^ → Hb(Fe^II^) + N_2_O_3_]. The second possibility assumes binding of nitrite to hemin, followed by combination with nitric oxide [Hb(Fe^III^)NO_2_^−^ + ^•^NO → Hb(Fe^II^) + N_2_O_3_]. In either case, MetHb is reduced to Hb. The two alternatives are associated with the problem of the precise binding site of nitrite, the iron or a porphyrin nitrogen. Interestingly, the T-conformation has a higher affinity to nitrite than the R-conformation [72]. Regardless of these details, N_2_O_3_ formation is obviously a biologically occurring process. However, the proportions by which the different RNS contribute in vivo to nitrosylation of proteins, thiols and aromates will require further investigation.

Another compound of especially high pathophysiological relevance is peroxynitrite, which is formed by an addition reaction of ^•^NO and the superoxide anion, O_2_^•−^. The resulting molecule (ONOO^−^) is not only a highly reactive compound, but also the source of strongly oxidizing and nitrating radicals. Upon protonation, the peroxynitrous acid undergoes a homolytic cleavage into ^•^NO_2_ and ^•^OH. The hydroxyl radical is a most strongly oxidizing ROS, but this decay of peroxynitrite is not favored with regard to the pK_a_. Otherwise, peroxynitrite would be practically inexistent in physiological fluids. Another route that may presumably be of even higher biological relevance, is initiated by the formation of an peroxynitrite-CO_2_ adduct, which also spontaneously decomposes [ONOOCO_2_^−^ → ^•^NO_2_ + CO_3_^•−^]. The resulting carbonate radical is not as reactive as ^•^OH, but still a strongly oxidizing compound, which undergoes many electron or hydrogen abstracting reactions otherwise known from the hydroxyl radical. Compared to ^•^OH, which is extremely short-lived, CO_3_^•−^ is resonance-stabilized because of its triangular symmetry and, thus, longer-lived and farther reaching.

The combination of elevated superoxide, ^•^NO and CO_2_ is particularly relevant under two conditions, mitochondrial impairment and hypoxia/hypercapnia [73,74]. In either case, mitochondrial electron leakage leads to enhanced production of O_2_^•−^. If mitochondrial dysfunction is related to inflammation or neuronal overexcitation, this is associated with elevated ^•^NO by iNOS or nNOS, respectively, which favors ONOO^−^ formation. In hypoxia/hypercapnia, ^•^NO is more strongly produced by eNOS, in attempts of enhancing blood flow. Under both conditions, ONOOCO_2_^−^ is easily formed, because mitochondria are the site of CO_2_ production and because reduced or interrupted blood flow causes hypercapnia. As a result, the CO_2_-adduct generates a mixture of ^•^NO_2_ and CO_3_^•−^. This leads to increased oxidative damage by CO_3_^•−^, and to increased nitration of protein-bound and free aromates by the combination of *NO_2_ and CO_3_^•−^, in which the latter free radical abstracts a hydrogen and, thus, facilitates ^•^NO_2_-binding [28]. Notably, aromate nitration via peroxynitrite is strongly stimulated by the presence of CO_2_, indicating a prevalence of the ONOOCO_2_^−^ decomposition over that of ONOOH [28].

## 3. Melatonin’s Oxidatively Formed Metabolites

**Figure 1:**
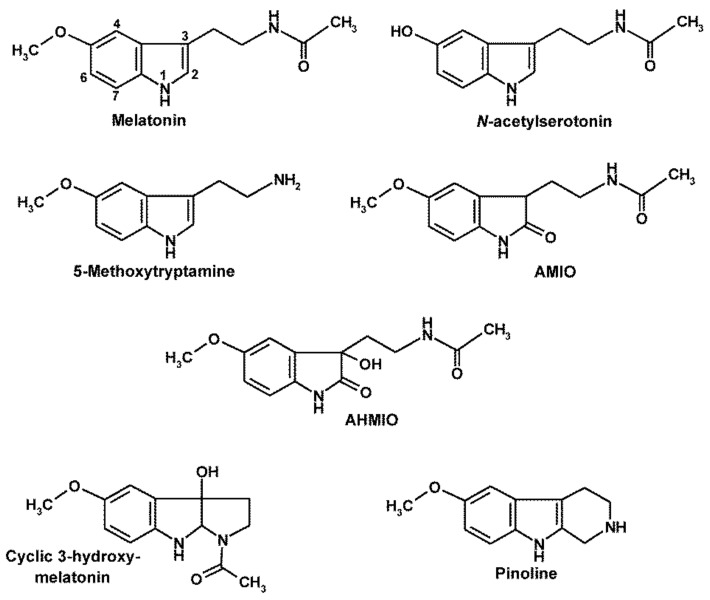
Schemes follow the same formatting. Melatonin, its hydroxylated metabolites and closely related analogs. Numbers at the melatonin molecule indicate nitrosylatable ring atoms and hydroxylatable carbon atoms. Abbreviations: AMIO, 3-acetamidoethyl-5-methoxyindolin-2-one; AHMIO, 3-acetamidoethyl-3-hydroxy-5-methoxyindolin-2-one.

Concerning the formation and elimination of RNS, the importance of melatonin is not restricted to this molecule but extends to several of its oxidatively produced metabolites. Melatonin (Figure 1) is oxidized by various enzymatic, preudoenzymatic and nonenzymatic processes [30,31,75,76]. With regard to the topic of this article, the indolic products are only briefly mentioned, because they either undergo reactions with RNS similar to those of melatonin or are poorly affected. A similarity of indole-typical nitrosylations at ring atom 1 (the pyrrole nitrogen) is found in several hydroxylated derivatives of melatonin, especially when ring atoms 4, 6 or 7 are concerned (cf. Figure 1). The same is reportedly or presumably the case in the deformylated and deacetylated metabolites, *N*-acetylserotonin and 5-methoxytryptamine, respectively, two compounds that can be both precursors and products in melatonin metabolism [76,77], and for secondary oxidation products of 5-methoxytryptamine [30,76]. Deviations in structure and chemical reactivity occur when melatonin is hydroxylated at ring atoms 2 or 3 (Figure 1). Hydroxylation at position 2 results in a compound that is tautomerically converted to an indolinone, with an equilibrium that is close to 100% on the ide of the latter. Therefore, the frequently read expression “2-hydroxymelatonin” is largely incorrect or, at least, strongly misleading. The recommended name is 3-acetamidoethyl-5-methoxyindolin-2-one (AMIO) [30]. An even stronger criticism is appropriate for a melatonin metabolite hydroxylated at both ring atoms 2 and 3. This is found in the literature as 2,3-dihydroxymelatonin, which falsely indicates a structure with a 5-bonded carbon atom (ring atom 3) that is chemically impossible. However, a previously formed indolinone structure allows hydroxylation at C3. Therefore, this compound should correctly be called 3-acetamidoethyl-3-hydroxy-5-methoxyindolin-2-one (AHMIO). However, this is only an intermediate that spontaneously turns into another important melatonin metabolite, *N*^1^-acetyl-*N*^2^-formyl-5-methoxykynuramine (AFMK), which is otherwise formed by various additional mechanisms [31]. Single C3-hydroxylation of melatonin leads to a molecular rearrangement to cyclic 3-hydroxymelatonin, a tricyclic compound (Figure 1) [78]. Notably, this molecule can also be converted to AFMK, however, by interaction with ROS [30,31].

A further tricyclic compound carrying an indolic core is pinoline (=6-methoxy- 1,2,3,4-tetrahydro-β-carboline), which had been related to melatonin (Figure 1). Pinoline had received its name from its presence in the pineal gland but can be also formed elsewhere. The precise mode of biosynthesis is still unclear, and potentially nonbiological formation in tissue extracts has been reported [79]. Pinoline is a potent free radical scavenger [80,81,82,83]. Similar to other β-carbolines, pinoline can be assumed to be nitrosylated [84] and to exert anti-nitrosylating reactions that protect important biomolecules such as DNA [85].

**Figure 2:**
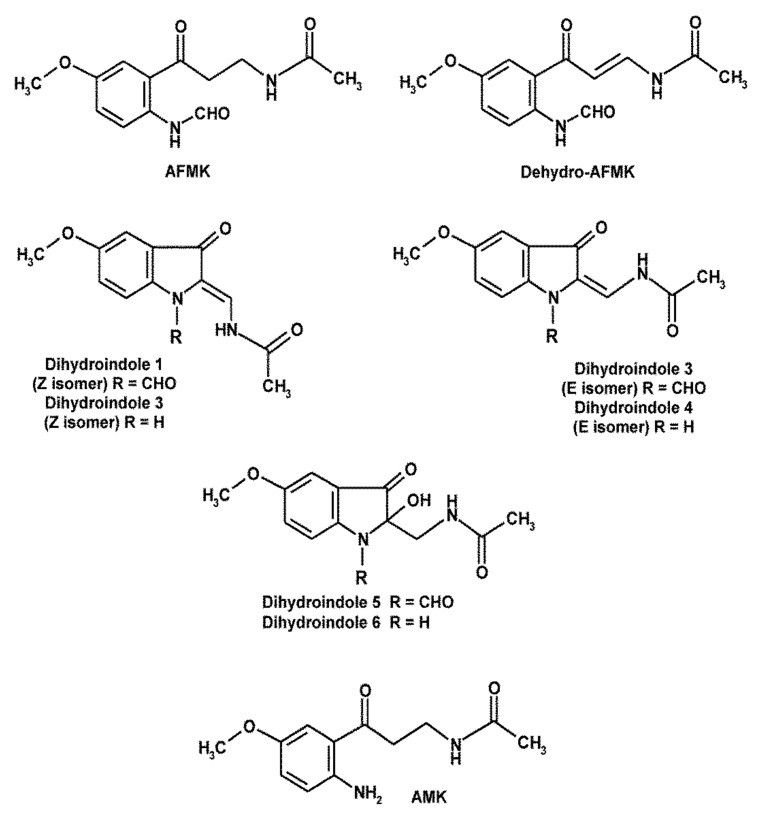
Melatonin-derived kynuramines and several secondary products with indolinone-like structures formed by interaction of AFMK with free radicals. Abbreviations: AFMK, *N*^1^-acetyl-*N*^2^-formyl-5-methoxykynuramine; AMK, *N*^1^-acetyl-5-methoxykynuramine; dihydroindoles 1, 2, *N*-(1-formyl-5-methoxy-3-oxo-2,3-dihydro-1H-indol-2-ylidenemethyl)-acetamide, Z and E isomers, respectively; dihydroindoles 3, 4, deformylated analogs of dihydroindoles 1 and 2; dihydroindole 5, *N*-(1-formyl-2-hydroxy-5-methoxy-3-oxo-2,3-dihydro-1H-indol-2-ylmethyl)-acet- amide; dihydroindole 6, deformylated analog of dihydroindole 5. Deformylated metabolites carry a nitrosylatable nitrogen.

Substituted kynuramines that originate by enzymatic, pseudoenzymatic or nonenzymatic oxidation of melatonin [30,31,75,76] are of special interest in terms of redox biochemistry, but also relevant to interactions with RNS and to the limitation of ^•^NO-dependent processes in inflammation and excitotoxicity. The primary kynuramine formed from melatonin or some of its oxidation products (cyclic 3-hydroxymelatonin and AHMIO) is AFMK (Figure 2). AFMK displays ROS-scavenging properties, especially with regard to the highly reactive ^•^OH, but to a smaller extent than melatonin [86,87]. With regard to various other free radicals, AFMK is much less reactive [86,87,88,89,90]. AFMK is an important intermediate in melatonin’s scavenger cascade, which is believed to strongly contribute to the overall efficacy of melatonin as a scavenger. This was first described for sequential elimination of 4 radicals [91] and later extended to the number of 10 [29].

Oxidation products deriving from AFMK have been mostly detected using chemical systems. Upon water radiolysis, dehydro-AFMK (Figure 2) was discovered as a novel compound [92]. Using the cation radical of ABTS [2,2′-azino-bis-(3-ethylbenzthiazoline- 6-sulfonic acid], ABTS^•+^, various other oxidation products were detected and some of them structurally identified [29], such as *N*-(1-formyl-5-methoxy-3-oxo-2,3-dihydro- 1H-indole-2-ylidenemethyl)-acetamide, present as Z- and E-isomers, and *N*-(1-formyl- 2-hydroxy-5-methoxy-3-oxo-2,3-dihydro-1H-indole-2-ylmethyl)-acetamide (Figure 2). Interestingly, the substituted kynuramine AFMK had rearranged to dicyclic compounds with 3-indolinone structures. Two further identified compounds were deformylated analogs [29] (Figure 2). This may be relevant, as these deformylated molecules should be accessible to N-nitrosylation at ring atom 1, whereas this cannot be expected for the depicted N-formylated substances. The presumably most important product deriving from AFMK is its deformylated analog, AMK (*N*^1^-acetyl-5-methoxykynuramine). Even though it has been often assumed that AMK may be formed within melatonin’s scavenger cascade, the direct evidence for this occurring in vivo has always been rather weak. In this author’s laboratory, in which melatonin and AFMK have been oxidized in numerous different chemical systems, AMK remained undetectable, as long as no redox-active enzymes were present. The oxidative formation of AMK from AFMK is typically based on 2-electron transfer reactions [31]. One reason for its poor detectability might be sought in the much higher reactivity of AMK relative to AFMK [86,87,88,89,90]. Therefore, any AMK formed in such systems may have disappeared soon after its eventual formation. The presence of deformylated oxidation products of AFMK [29] may indicate that AMK may have been an intermediate, although the possibility remains that the formylated 3-indolinones are deformylated after the secondary cyclization. When AMK was directly oxidized in chemical systems, short-lived products appeared that could not be structurally characterized because of their instability [32], but which were never detected during chemical AFMK oxidation [30]. Nevertheless, AFMK is converted to AMK by enzymes, such as hemoperoxidases (including catalase) and arylformamidases [31]. Moreover, AFMK can be photochemically deformylated to AMK by UVB/C by releasing CO [93]. Whatever the main processes of AMK formation are, this compound has been shown to be present in biological material [25,26,27,90,94,95]. Additionally, the latter study demonstrated the formation of hydroxy-AMK and its glucuronide conjugate [95], indicating an active AMK clearance. The hydroxylation of AMK occurred at the aromatic ring, but the precise position had not been identified. The physiological occurrence of AMK is of potentially higher importance, because of its high reactivity to several ROS, in particular, ^•^OH [87,88,89], which can even exceed that of melatonin in aqueous media [88], to singlet oxygen, again with higher affinity than melatonin [96], and to CO_3_^•−^ [28,86,89], as well as to various interactions with RNS and because of ^•^NO diminishing actions, which will be discussed in Section 5.

## 4. Nitrosylation, Nitration and RNS-Mediated Oxidation of Melatonin and Its Metabolites

**Figure 3:**
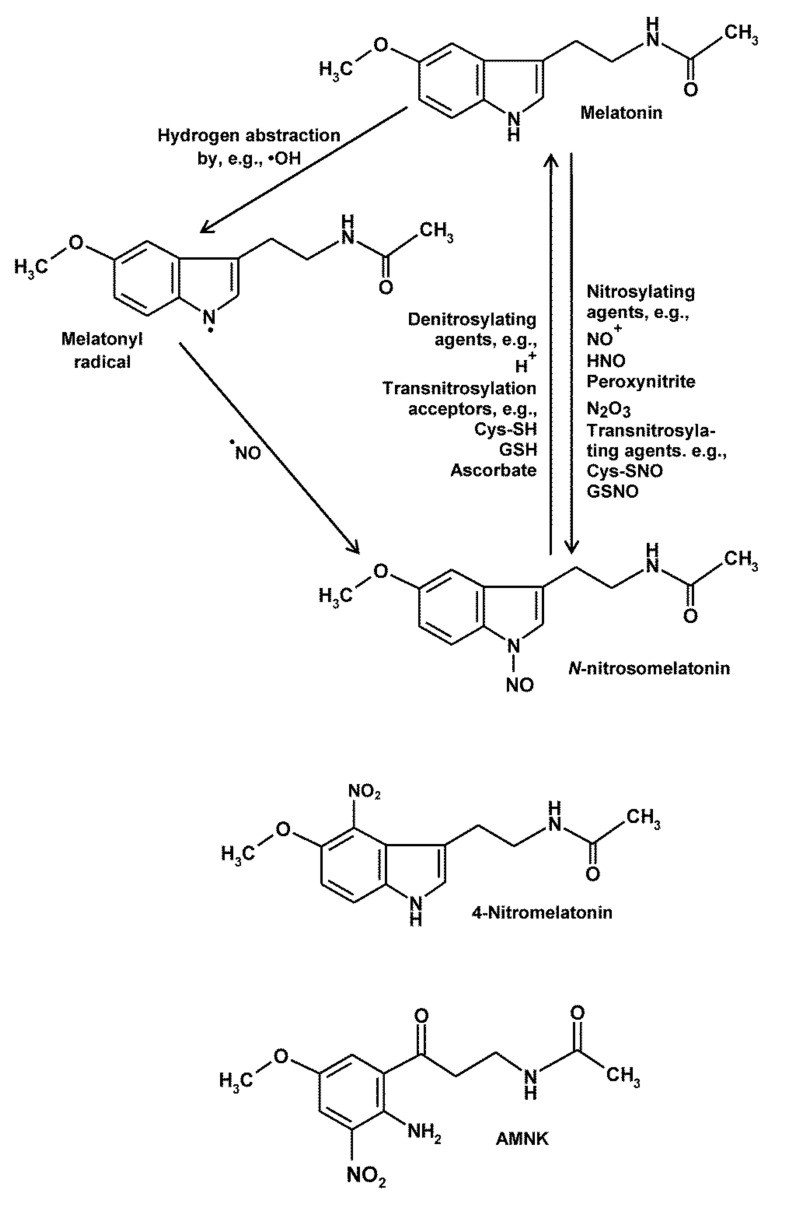
Nitrosylation of melatonin at nitrogen (ring atom 1), denitrosylation of *N*-nitrosomelatonin and nitrated products of melatonin and AMK. Abbreviation: AMNK, *N*^1^-acetyl-5-methoxy-3-nitrokynuramine. Additional nitrosylations of melatonin are possible at carbon atoms indicated in Figure 1.

Nitrosylation of melatonin has been documented most extensively for the ring atom 1, the nitrogen in the pyrrole ring [9,68,69,97,98,99,100,101,102,103,104,105,106,107]. C-nitrosylation has been also found to be likely at ring atoms 2, 4, 6 and 7 [9] (cf. Figure 1), but the biological relevance of these products has not been sufficiently studied. Various nitrosylating compounds have been shown to give rise to *N*-nitrosomelatonin (= 1-nitrosomelatonin). The interaction with the most abundant RNS, ^•^NO, would require hydrogen abstraction from melatonin, e.g., by oxidizing free radicals, which allows combination of the resulting neutral melatonyl radical with ^•^NO [99] (Figure 3). However, several alternatives exist. The interaction with the easily nitrosylating redox congener NO^+^ leads to the same product but is possible with melatonin directly and yields H^+^ release [99] (Figure 3). With regard to the extremely short half-life of NO^+^ in aqueous solution (about 10^−10^ s at pH 7.4) [35,56], this chemically easily possible mode of action should be physiologically less important. Of higher relevance are presumably reactions with peroxynitrite, HNO and, if formed at sufficient levels, N_2_O_3_; moreover, transnitrosylation from other compounds such as nitrosothiols (e.g., *S*-nitrosoglutathione, *S*-nitrosocysteine) or *N*^1^-nitrosotryptophan seem to be of importance.

However, *N*-nitrosomelatonin has been shown to redonate nitric oxide [103,108,109,110,111,112]. This can happen by releasing ^•^NO, though in a relatively slow process [109,110]. Whether this happens only in a homolytic process that would generate a melatonyl radical remains to be clarified. Another reaction seems to consist in the release of NO^+^, which would be possible by protonation at *N*^1^ (Figure 3). This reaction is supported by the observation of enhanced denitrosylation upon acidification [110]. Moreover, *N*-nitrosomelatonin is able to transnitrosylate suitable compounds such as thiols, e.g., reduced glutathione or cysteine, other indoles including tryptophan and tryptophan side chains in proteins and also ascorbic acid [105,110,111,112].

Melatonin’s property of being reversibly nitrosylated raises the question of its biological relevance. As far as *N*-nitrosomelatonin redonates ^•^NO, the balance of zero does not seem to allow conclusions on protection from nitrosative stress. However, two aspects may lead to a different view. First, if melatonin has been nitrosylated by an RNS such as HNO or peroxynitrite and, thereafter, releases NO^+^, the extremely short half-life of the nitrosonium cation [35,56] may shift the balance. Even though this half-life that is shorter than that of a hydroxyl radical indicates an extremely high reactivity, damage by NO^+^ is usually rather modest at physiological concentrations, because this compound mainly reacts with OH^−^ that is amply abundant in water (NO^+^ + OH^−^ → HNO_2_) and hits biomolecules at relevant rates only at high concentration. The second aspect concerns the possibility of displacement. As redonation of ^•^NO from *N*-nitrosomelatonin is a rather slow process [110], the possibility remains that the scavenged ^•^NO is displaced from a site of potential damage to a site of release where ^•^NO is less likely to undergo adverse reactions. Even though this aspect has not been sufficiently studied in terms of protection in animals, displacement of *N*-nitrosomelatonin has become an interesting topic in plant physiology, in which this compound has been discussed as a long-distance carrier of nitric oxide [104,107].

The nitrosylation reactions described for melatonin are also possible with various other indoles, though with differences in affinities and rates. *N*-acetylserotonin, serotonin, 5-hydroxytryptophan and *N*-acetyltryptophan exhibited similar reactions as melatonin, however, at lower scavenging rates [97,102,113]. These compounds were poorly reactive toward ^•^NO but were correspondingly nitrosylated by other RNS or by combinations of ^•^NO and ROS [114]. Due to their structural similarities, hydroxylated metabolites of melatonin and β-carbolines should also be able to undergo nitrosylation reactions, what has to date been insufficiently studied. In the case of β-carbolines, the observed protection against DNA nitrosylation [85] may be in line with this assumption. Reactions of tetrahydro-β-carbolines with nitrite-based nitrosylating reagents had been investigated, but the nitroso-products were unstable and turned into dihydro-β-carbolines [84]. Unfortunately, pinoline, the β-carboline with highest similarity to melatonin has not been studied in this regard and the comparisons to melatonin remained restricted to antioxidant activities [80,81,82,83], although nitric oxide-induced peroxidation reactions [83] may have been accompanied by pinoline nitrosylation. Nitrosylation of melatonin-derived or other kynuramines has been only thoroughly studied in the case of AMK. However, the respective results strongly deviated from those in melatonin or other indoles and will, thus, be treated separately in Section 5.

Nitration of indolic compounds, which leads to another category of RNS-derived products, has been much less investigated than nitrosylation. 6-Nitrotryptophan and *N*-acetyl-6-nitrotryptophan were identified as products of reactions with peroxynitrite [115]. However, the outcome concerning melatonin nitration by peroxynitrite remained rather modest. One study reported the absence of stable nitration products [116], whereas two others detected minor amounts of 4-nitromelatonin [98,115] (Figure 3). Most melatonin metabolites have not yet been tested with regard to nitration. To date, a stable nitration product has been only observed in the case of AMK, which was converted to *N*^1^-acetyl-5-methoxy-3-nitrokynuramine (AMNK) [28] (Figure 3). Quantifiable amounts of this product were mostly produced in the presence of peroxynitrite and bicarbonate as a CO_2_ source. With peroxynitrite alone, no substantial quantities of this compound were detected, indicating that the protonation of peroxynitrite that is followed by cleavage into ^•^OH and ^•^NO_2_ is insufficient for this reaction. Nevertheless, generation of ^•^OH would have led to some AMK oxidation [88,89], but the products had either been too unstable to allow interactions with ^•^NO_2_ or the rates of peroxynitrite protonation had remained too low. However, the ONOOCO_2_^−^ adduct that decomposes into CO_3_^•−^ and ^•^NO_2_ is obviously more appropriate for nitration of such aromates. Therefore, a non-canonical nitration by free radicals explains best the chemical process [28], in which AMK is first oxidized by CO_3_^•−^, as known from other experiments [88,89], followed by combination of the hydrogen-abstracted free radical with ^•^NO_2_. This interpretation is supported by observations of CO_2_-enhanced oxidation reactions of peroxynitrite [117] as well as by comparable studies on the pathophysiologically important tyrosine nitration [18,22,118], in which nitrotyrosine formation by peroxynitrite was strongly enhanced by CO_2_/HCO_3_^−^ and researchers arrived at the same conclusion [119,120,121]. In this regard, the efficacy of ONOOCO_2_^−^ in nitrating AMK contrasts with a remarkable paucity observed with melatonin. Even though melatonin can be oxidized by peroxynitrite or hydroxyl radicals deriving from peroxynitrous acid, and although melatonin is easily oxidized by carbonate radicals [74,88,89,122], the resulting metabolite is typically AFMK, which is formed either directly or indirectly via cyclic 3-hydroxymelatonin or AHMIO. However, AFMK is poorly oxidized by CO_3_^•−^, contrary to the highly reactive AMK [88,89]. Therefore, a radical that can combine with ^•^NO_2_ is not formed from AFMK, which is anyway the considerably more stable metabolite. Apart from the details concerning melatonin, AFMK and AMK, the important lesson from the results obtained with ONOOCO_2_^−^ is that oxidation and nitration can be generally associated in chemical systems, not just physiologically via inflammation, and that the levels of CO_2_ generally matter in redox physiology, whether at the mitochondrial level, i.e., in the CO_2_-producing organelle or under conditions of hypercapnia such as in ischemia or respiratory insufficiency [74].

## 5. Peculiarities of AMK

**Figure 4:**
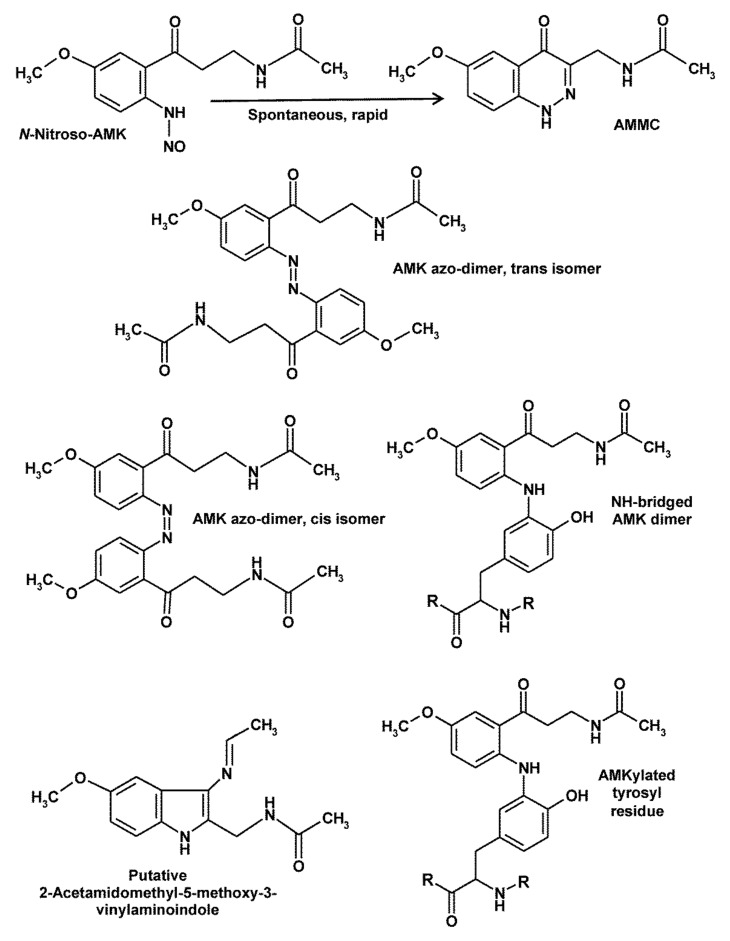
Nitrosylation of AMK to AMMC and oxidative products (dimers, putative vinylaminoindole, tyrosyl adduct) of AMK. Abbreviation: AMMC, 3-acetamidomethyl-6-methoxy-cinnol- inone. The structure of 2-acetamidomethyl-5-methoxy-3-vinylaminoindole is still hypothetic, but consistent with high-resolution MS [127]. Dimerization is indicative of AMK’s potential for nitrogen-dependent adduct formation, as in the interaction with tyrosyl residues. The similarity of the vinylaminoindole to melatonin would suggest possible *N*-nitrosylation as known for melatonin.

AMK exhibits several differences to its precursor, AFMK and to melatonin, too, especially concerning ROS and RNS interactions. As will be shown, redox behavior and reactions with RNS can be interrelated under several conditions. As discussed above, AMK is, compared to AFMK, considerably more reactive to some oxidizing free radicals, in particular to ^•^OH and CO_3_^•−^, and also to singlet oxygen. This difference is even more pronounced with regard to RNS. The unusually high reactivity towards such agents was discovered by chance, when AMK was found to be converted on dry silica chromatography plates by trace amounts of RNS present in normal air (in a room apart from the laboratory). Among the three products, one was identified as the nitration product AMNK, the second one as *N*-[2-(6-methoxyquinazolin-4-yl)-ethyl]-acetamide (MQA) and, the third as 3-acetamidomethyl-6-methoxycinnolinone (AMMC) [28]. MQA was formed via oxidatively mediated (in air: by ozone?) interaction with ammonia [123]. In biological material, an interaction with carbamoyl phosphate appeared to be a likely mechanism [123], and MQA formation was also observed in yeast incubated with AFMK [124]. More importantly, AMMC was identified as a product formed by nitrosylation [28,125]. Detailed studies showed that AMMC is nitrosylated at the anilinic nitrogen, a structure that cyclizes to a substituted cinnolinone (Figure 4) [36,125]. Contrary to *N*-nitrosomelatonin, which redonates nitric oxide, AMMC is rather stable and decays only in the presence of strong oxidants such as ^•^OH. No enzymatic catabolism of AMMC is known to date. When testing AMMC formation from AFMK, this was only observed upon combined incubation with the ^•^NO donor PAPA-NONOate and H_2_O_2_ for extended periods of many hours, contrary to the rapid conversion of AMK with the ^•^NO donor [36]. Importantly, all three NO congeners, NO^+^, ^•^NO and HNO were similarly efficient in nitrosylating AMK to AMMC [36], as was later also reported for N_2_O_3_ [106] and will presumably occur with other nitrosylating agents, too. The different reaction pathways with the three congeners have been published elsewhere [36].

Other peculiarities of AMK became evident in experiments on oxidation of this compound, which also shed light on the reactivity of its anilinic nitrogen under oxidative conditions. When oxidized by ABTS cation radicals (ABTS^•+^), for purposes of using a free radical that does not easily destroy products to be analyzed, several oligomers of oxidized AMK intermediates were found and structurally characterized [126]. Three dimers are depicted in Figure 4. Two of them are azo dimers, whereas, in the third one, the anilinic nitrogen is attached to ring atom 3 of the second AMK residue. For additional trimeric or tetrameric compounds see ref. [126].

Oxidation of AMK in other reaction systems using more aggressive radicals turned out to be affected by rapid destruction of products. Attempts of circumventing this problem were made by combining ABTS^•+^ with H_2_O_2_, to allow a primary AMKyl radical to interact with another ROS instead of a second AMKyl radical or AMK derivative. These experiments revealed several new products, among which a prominent compound was characterized by high-resolution mass spectrometry (mass 259.1393410; molecular formula C14H17N3O2), absorption spectrum (UV/Vis) and fluorescence properties [127]. Even though the structure was not unambiguously identified, the difference to AMK (product: plus 2 C, minus 1 O, plus 1 N) indicated transfer of an acetamido group from one AMK to another AMK, thereby replacing a double-bonded oxygen by a vinylamino group, presumably at C3′, with the additional possibility of secondary cyclization to a 2-acetamidomethyl-5-methoxy-3-vinylaminoindole [127]. Again, the acetamido/vinyl- amino exchange underlines the particular property of AMK in donating and accepting nitrogen-containing residues.

The reactivity of AMK is also obvious in another process that involves its anilinic nitrogen. In studies concerning the interaction of AMK with aromatic amino acids, it produced adducts with both tyrosine and tryptophan. While the structures of tryptophan adducts were difficult to analyze because of polymerizations that occur under in vitro conditions, the binding of AMK to tyrosine was characterized by using tyrosine’s side chain fragment, 4-ethylphenol [128]. This type of adduct should not only be possible with free tyrosine, but also with tyrosyl residues in proteins (Figure 4). As the reactive *N*^2^ of AMK is attached to ring atom 3 of tyrosine, this would prevent 3-nitrotyrosine formation. The biological significance of protein AMKylation [128] would still require further clarification. This should have consequences especially in tyrosine-rich domains of proteins, e.g., the cytosolic domains of receptor tyrosine kinases and non-receptor tyrosine kinases, where autophosphorylation and attachment of phosphotyrosine binding (PTB) domain-containing proteins might be inhibited. In fact, antiproliferative actions of AMK have been observed in keratinocytes and melanoma cells [26], effects that may contribute to previously observed antiproliferative properties of melatonin in the skin [25]. Notably, no receptor is known for AMK and the inhibition may, thus, be receptor-independent. Moreover, the kinetics of these inhibitory effects is highly unusual. Inhibition was already observed with concentrations as low as 10^−12^ and 10^−10^ M, but higher levels did not lead to half-maximal inhibition within a meaningful range. This type of dose dependency is strongly reminiscent of data on nNOS [15]. Again, inhibition by AMK was observed in the range of 10^−11^ M, but half-inhibition required considerably higher levels. The precise reasons for these atypical inhibition kinetics remain to be clarified. However, protein AMKylation may explain effects in the low concentration range, whereas limited abundance of AMKylatable sites in the respective proteins may not allow stronger inhibitions at higher concentrations. Concerning the phyiological relevance of such AMK effects, the paucity of data concerning AMK levels in tissues remains to be a problem. Blood levels may be irrelevant, if AMK is mainly formed from tissue melatonin. Moreover, in many cell types, the highly reactive AMK may disappear too soon to allow judgments on the effects of this substance that readily decays and is only transiently present. However, the skin seems to represent an exception, in which AMK was detected in levels of 1.5 ng/mg protein (Afroamericans) or 0.5 ng/mg protein (Caucasians). The ethnic differences may be related to the potent singlet oxygen scavenging by AMK, which is even stronger than that by melatonin [96]. More data on AMK levels in other tissues and effects on further tyrosine-rich proteins would be highly desirable.

## 6. Pathophysiological Relevance of Actions against Nitration, RNS-Mediated Oxidation and Nitrosylation

Modification or proteins and other biomolecules by RNS or their derivatives can lead to severe dysfunctions of metabolism. This concerns especially proteins of mitochondrial complexes, as will be discussed in the subsequent Section 7. Moreover, the appearance of nitrated proteins can be regarded as a sign of severe stress by combined actions of RNS and ROS, as they occur when peroxynitrite is protonated or forms CO_2_ adducts, followed by cleavage into oxidizing radicals and ^•^NO_2_. This is observed in various different pathologies [129,130,131,132,133,134,135], such as cardiovascular [136,137,138,139] and neurodegenerative diseases [140,141,142,143,144,145], but also under conditions of ischemia/reperfusion [146,147,148,149,150,151,152]. In addition, mutagenic and carcinogenic effects occur, when DNA is hit by ROS and RNS, especially peroxynitrite: DNA bases are modified by RNS that cause nitration (e.g., formation of 8-nitroguanine) [153,154,155,156,157], deamination (e.g., xanthine) [85,158] or both (e.g., 8-nitroxanthine) [159]. Nitrated purine bases such as 8-nitroguanine and 8-nitroxanthine are a potential source of DNA instability because of spontaneous depurination that leads to apurinic sites [159]. As far as nitration reactions are based on free radicals formed from peroxynitrite, the simultaneously produced oxidizing radicals, ^•^OH or CO_3_^•−^, can also act independently of ^•^NO_2_ and modify proteins and nucleic acids oxidatively or disturb biological systems by lipid peroxidation. In these cases, the multiply demonstrated antioxidant actions of melatonin are relevant and often effective in protecting biomolecules and cellular functions that depend on them [1,2,10,11,12,13,20,21,22]. The extent of contributions by melatonin metabolites, in particular, cyclic 3-hydroxymelatonin and AMK, remain to be clarified with regard to the quantities formed under the diverse conditions. In any case, the regulatory functions of both melatonin and AMK in reducing ^•^NO synthesis [14,15,16,17,21,22,23] can be expected to diminish peroxynitrite-induced damage, in addition to attenuation of ^•^NO-mediated inflammatory responses that cause release of oxidants and additional ^•^NO formation.

By contrast with nitrative modifications of proteins and nucleic acids, which may generally be judged to be unfavorable and disease promoting, another RNS-mediated modification, namely, protein S-nitrosylation, can lead to either adverse or physiologically required effects. Adverse actions can be expected when key enzymes are inappropriately blocked by such processes. S-nitrosylation may contribute to interruptions in electron transport chains, although ^•^NO binding to heme and non-heme irons may be at least equally or more important [160]. Among the important and physiologically desired actions, mechanisms of reversible S-nitrosylation can determine the activities of key enzymes in a controlled way as part of signaling routes. Numerous proteins are meanwhile believed to be controlled in this way [161]. Moreover, heme binding of ^•^NO and S-nitrosylation of cysteine residues can interact and be part of on/off switching mechanisms. For instance, soluble guanylate cyclase (sGC) is activated by binding of ^•^NO to Fe^II^ in heme. Oxidation to Fe^III^ leads to an inactive state, but an ^•^NOFe^III^ complex dissociates into NO^+^ and Fe^II^; the released NO^+^ S-nitrosylates a specific cysteine residue of sGC, which causes a conformational change that desensitizes the sGC to ^•^NO; the sGC needs to be denitrosylated to regain sensitivity to ^•^NO [162]. This example from the physiologically and pathophysiologically important control of vascular smooth muscle relaxation sheds light on two aspects, (a) the necessity of also considering intramolecular nitrosylation and (b) the relevance of denitrosylating processes. It is necessary to be aware of the multiple possibilities of transnitrosylation. S-nitrosylated compounds (SNOs) of low molecular weight, such as *S*-nitrosocysteine (CysSNO) or *S*-nitrosoglutathione (GSNO) can easily transfer NO to cysteines in various proteins and vice versa. Additionally, SNO proteins can undergo transnitrosylation reactions with other proteins, Moreover, several enzymatic systems are specialized for protein denitrosylation, such as GSNO reductases, SNO-CoA reductases and thioredoxin-related denitrosylases, for which numerous target proteins are known [163].

Apart from the above-mentioned pathologies in which nitrative modification and damage to proteins and nucleic acids are involved in the respective etiologies and secondary damage such as cell death and losses of neuronal connectivity, nitrosylation is also of relevance to numerous other diseases. In addition to effects on immune cells and mitochondrial functionality, which shall be discussed in the subsequent Section 7, S-nitrosylation of proteins seems to be a frequent cause of pathological dysregulation [163]. In cancer, S-nitrosylation of regulatory proteins has been shown to drive disease onset, progression and treatment resistance [164,165,166,167,168,169,170], to favor metastasis via epithelial to mesenchymal transition, migration/invasion [171,172] and also to promote tumor growth by inducing angiogenesis [172]. NOS upregulation, often by several tissue or cancer type-specific mechanisms, has been repeatedly found to be a decisive process in tumor development and properties [169,171,172,173,174,175,176], but the levels of transnitrosylating nitrosothiols may be even more important than the mere ^•^NO levels [177], although their elevation is a precondition of nitrosothiol formation. The role of protein S-nitrosylation is increasingly perceived in its importance to cancer research. A complete record would go far beyond the scope of this article. However, it seems necessary to direct the readers’ attention to several points that may easily be judged to be highly controversial. For instance, upregulation of iNOS via transcription factors such as Ets-1 and resulting elevated ^•^NO levels have been found to be decisive parts of oncogenic signaling networks [176,177,178,179]. However, in other studies, opposite effects were obtained. In a glioma cell line, S-nitrosylation of ERK1/2 prevented ERK1/2 phosphorylation and, thus, inhibited cell growth [180]. In colon and mammary cancer cells, nitrosylation of TNFα and cIAP1 (cellular inhibitor of apoptosis protein 1) caused a switch from cell survival to cell death by assembly of a death complex [181]. In lung cancer cells, S-nitrosylation of the H_2_O_2_-eliminating enzyme Prdx2 (peroxiredoxin-2) led to increases in H_2_O_2_ levels, which activated AMPK, followed by phosphorylation of SIRT1 and, thus, abolishment of its deacetylating activity. As a result, cells were driven into apoptosis [182]. Collectively, these results are certainly opposed, but not necessarily contradictory. The divergence of cancer cells has to be taken into consideration, and the decisive differences concern the factors that are preferably nitrosylated. Moreover, the findings on SIRT1 in lung cancer cells are indicative of another contrasting field, which is additionally related to melatonin. In these cancer cells, active SIRT1 is obviously anti-apoptotic and, thus, cancer promoting [182]. While melatonin typically upregulates SIRT1 in the majority of nontumor cells [183], the opposite was found in prostate cancer cells [184]. This discrepancy is explained by the fact that SIRT1 is an accessory factor in the cellular circadian oscillator machinery and that cancer cells have to silence those circadian oscillator components that possess tumor suppressing properties [183,185,186,187]. As a consequence, the oscillator is dysregulated and two of its components that promote cell survival and proliferation, SIRT1 and CLOCK, are upregulated. Under this condition, melatonin gradually normalizes SIRT1 expression, perhaps by influencing the oscillator via its chronobiotic properties. The opposite effects of melatonin in tumor and nontumor cells extend to other functions, especially regarding the control of apoptosis. In nontumor cells, melatonin has been shown to act mostly as an anti-apoptotic agent, whereas it is often proapoptotic in cancer cells [183,188,189].

S-nitrosylation of proteins has also been shown to promote in multiple ways neurodegenerative processes [190,191,192,193,194,195,196,197,198,199,200,201,202,203,204,205,206]. Of course, considerable differences exist between the various pathologies, depending on the disease-specific dysregulated functions. In this context, it seems important to discriminate between the target proteins that are S-nitrosylated. Some target-related examples shall be mentioned. If S-nitrosylation keeps DRP1 in the state of an active GTPase, this results in enhanced mitochondrial fission, with consequences of permeability transition, enhanced ROS formation and cell death [207,208,209]. If a protein phosphatase is inhibited by S-nitrosylation, the imbalance towards the phosphoprotein side causes another type of dysregulation that may also end up in neurodegeneration [210]. Another detrimental type is stress by enhanced protein misfolding via S-nitrosylation of chaperones [196,211,212,213]. Comparable considerations should apply to many other diseases in which dysregulation by S-nitrosylation has been observed, such as metabolic syndrome [197,212,213,214,215,216] and related pathologies, e.g., cardiovascular diseases [217,218] and diabetes [216,218,219,220,221,222,223,224], in nephropathies [221,225], hepatic pathologies [212,213,226], cardiomyopathies [218], neuromuscular dysfunction [227], and disturbed mesenchymal stem cell differentiation in osteological pathologies [228].

With regard to the widespread importance of S-nitrosylation that causes inhibition or activation of countless proteins of central importance to cell functions or pathologies, there is good reason to assume that the inhibitory actions of melatonin and AMK on ^•^NO synthesis contribute to the protective actions exerted by these regulators. The extent of these effects relative to other beneficial actions remains to be clarified. In many studies, beneficial effects of melatonin have been only attributed to its antioxidant properties, which were discovered prior to its ^•^NO-attenuating actions. The meanwhile broader understanding of the roles of protein S-nitrosyls and the underlying mechanisms of nitrosylation and denitrosylation reveal another requirement for further studies on melatonin and its metabolites as modulators of S-nitrosyl modifications. Even though several forms of protein denitrosylases are known that use different categories of cofactors [161], the possibility that beneficial effects of melatonin might include the enzymatic reversal of unfavorable S-nitrosylations has not yet attracted the attention of melatonin researchers. This gap should soon be closed, also with regard to the nexus with redox systems. In particular, components of high importance to mitochondria are involved in main denitrosylation mechanisms, such as glutathione, coenzyme A and thioredoxins. The important earlier work on maintenance of the GSH/GSSG ratio [17,229,230,231,232,233] may indicate an influence on the S-nitrosylation status. The availability of HS-CoA and, indirectly, SNO-CoA should be related to the citrate cycle throughput. The relationship between melatonin and thioredoxins has still been widely neglected, although these appear to be of highest importance to H_2_O_2_ elimination, which has to date been mostly studied on the basis of glutathione peroxidases and catalase. The influence of melatonin on a thioredoxin system has been recently reported [234] but does not yet allow conclusions concerning denitrosylation.

## 7. Consequences to Inflammation and Mitochondrial Integrity

Within the complex of nitrative, nitrosative and oxidative damage by RNS, the role of inflammation as a source of reactive intermediates and the consequences to mitochondria are of utmost importance. Anti-inflammatory actions of melatonin have become more and more apparent during the last years, although earlier assumptions had focused on the proinflammatory potential of this agent. This shall not be discussed here in all details, because this topic was addressed in several recent reviews [22,23,225,226,227,228,229,230,231,232,233,234,235,236,237,238,239,240,241,242]. Instead, a few decisive findings shall be highlighted. The anti-inflammatory potential of melatonin becomes evident by the repeatedly demonstrated prevention of inflammasome activation, which has been observed under various conditions [236,239,243,244,245,246,247,248,249,250,251]. Moreover, relationships have emerged between melatonin and proinflammatory microRNAs [239,252], some of which were recently shown to be downregulated in conjunction with the NLRP3 inflammasome [253]. Another presumably important aspect concerns the involvement of sirtuins in melatonin’s anti-inflammatory actions. The mediation of melatonin’s effects by especially, but not exclusively, sirtuin 1 (SIRT1) has emerged during the last years and gained increasing attention [183,186,235,238,239,245,252,253,254,255]. The mediation of melatonin effects by SIRT1, which was blocked by sirtuin inhibitors and *Sirt1* siRNA, gave rise to the concept of an extended melatonin signaling, which goes beyond the primary MT_1_/MT_2_ signaling pathways [256]. In the context of inflammation, substantial effects by SIRT1 have been reported, which exceed those reported up to now for melatonin and which may either complement the actions by melatonin and/or transmit melatonin’s messages. Details concerning the various factors regulated dependently or in parallel have been summarized elsewhere [23,235,239]. A further detail concerning the pro- versus anti-inflammatory balance may have been poorly considered in the past, but could be of special importance, namely, the influence of melatonin on macrophage polarization. Melatonin favors the anti-inflammatory type M2 at the expense of the proinflammatory type M1 [257]. As this M1/M2 polarity also exists in microglia, a corresponding influence by melatonin should be highly relevant to brain inflammatory conditions and their treatment. The remarkable anti-inflammatory potential of melatonin is particularly evident in its suppression of sepsis and other high-grade inflammatory diseases. After the seminal articles on protection against experimental sepsis [16,17,19,229,258,259], numerous other cases have been described concerning bacterial and viral infections in animals and humans, in which melatonin turned out to provide efficient treatment, as summarized elsewhere [260,261,262,263,264,265,266,267,268]. Of course, the anti-inflammatory actions of melatonin comprise several different mechanisms [23], which, however, complement each other. Nevertheless, the overproduction of ^•^NO, especially in conjunction with enhanced O_2_^•−^ formation because of high mitochondrial electron leakage or NAD(P)H oxidase activities, and with elevated CO_2_ levels, seems to be a crucial combination that drives oxidative, nitrosative and nitrative damage and initiates vicious cycles. These comprise the formation of DAMP (damage-associated molecular pattern) factors, activation of toll-like (TLR) and other DAMP-controlled receptors such as RAGE (receptor of advanced glycation end products), and initiation of cytokine storms [23,253,255,265]. These processes are inhibited by melatonin at multiple stages and also include actions by SIRT1 [23,235]. Especially under the aspect of inflammatory vicious cycles, the limitation of ^•^NO synthesis appears to be a key property of melatonin that prevents the self-stimulating overproduction of RNS. This is mainly based on the inhibition of iNOS and nNOS [14,15,16,17,229,258,259]. Additional protective actions by melatonin, including scavenging of RNS and ROS and various other mitochondrial effects, may be seen as supporting and complementing the anti-inflammatory arsenal, but may not suffice without NOS inhibition. To what extent melatonin metabolites, especially AMK, contribute to the limitation of ^•^NO synthesis, has not yet been sufficiently studied under in vivo conditions. However, inhibitions by AMK observed at concentrations in the 10^−11^ molar range [15,26] let this possibility appear not entirely unrealistic. With regard to such actions, more specifically on iNOS [269,270], and also to the physiological and, perhaps, toxicological properties of AMMC formed from AMK by scavenging of ^•^NO, more in vivo studies are required.

## 8. Conclusions

Melatonin is an agent that is ubiquitously present in the entire body. In terms of quantity and often also of concentration, its amounts exceed the circulating levels, sometimes by far. Moreover, exogenous melatonin can be administered at even higher doses to attain therapeutic concentrations [4,266,267]. Based on these conditions, melatonin appears as a highly suitable compound for counteracting pathologies associated with detrimental elevations of RNS. These comprise various diseases with etiologies in which low- or high-grade inflammation is a crucial driving factor, such as neurodegenerative diseases, ischemia/reperfusion and sepsis. Notably, the RNS-promoted damage to molecules, cells and tissues is typically associated with oxidative stress. Three main causes can be identified for being responsible for this association: (i) ^•^NO-activated inflammation that results in the release of cell damaging oxidants, (ii) impairment of mitochondrial function by RNS, which leads to increased electron leakage, and (iii) the formation of peroxynitrite by combination of the RNS ^•^NO and the ROS O_2_^•−^, which further results in peroxynitrite-derived radicals such as ^•^OH, CO_3_^•−^ and ^•^NO_2_. As melatonin is not only a highly potent antioxidant that additionally activates cellular antioxidative machineries, but also interferes with RNS production, it exhibits a unique combination of properties for preventing both oxidative and nitrosative/nitrative damage.

Another important feature of melatonin results from its metabolism, which is characterized by another uniqueness, namely, being source of a cascade of numerous metabolites with protective properties. Even though many of these compounds that are depicted in this article have been poorly investigated with regard to their pharmacological properties, a look at their structures reveals that all those that do not contain a formylated nitrogen should be capable of interacting with ^•^NO and its redox congeners. This is best understood in the case of AMK, from which the stable compound AMMC is formed by nitrosylation [28,36,125]. Moreover, AMK shares other properties with melatonin, such as scavenging of oxidizing free radicals [31,88,89] and singlet oxygen [96], downregulating iNOS and nNOS [14,15] as well as cyclooxygenase-2 (COX-2) [270]. Jointly, melatonin and AMK may, thus, attenuate inflammatory responses. Such a role of AMK may deserve further investigation, but its effects have been demonstrable down to very low concentrations of 10^−10^–10^−12^ M. When reinvestigating these effects, the apparently low concentrations of AMK in many tissues should not be misinterpreted, since AMK, when produced, readily disappears because of its high reactivity toward RNS and ROS [31].

With regard to the roles of melatonin and AMK, two additional aspects are worth further investigation: (i) While the ^•^NO-scavenging properties of both compounds are well understood and should also contribute to reductions of undesired protein S-nitrosylation, the recently emerged mechanisms of physiological S-denitrosylation [161] should be studied with regard to the influence of melatonin. As melatonin has been shown to influence GSH levels and also play important roles in mitochondria, additional effects of melatonin on the three major types of S-denitrosylases, i.e., GSNO reductases, SNO-CoA reductases and thioredoxin-related denitrosylases, would not be a surprise. (ii) The effects of extremely low AMK levels deserve in-depth studies concerning AMKylation of protein tyrosines. Especially with regard to the regulatory importance of these amino acid residues in tyrosine-rich proteins such as receptor and non-receptor tyrosine kinases, this type of modifications might attract considerable interest. The observed inhibitory effects of AMK on cell proliferation [26,27] are, at least, encouraging.
